# Robot-as-a-Service: From Cloud to Peering Technologies

**DOI:** 10.3389/frobt.2021.560829

**Published:** 2021-05-20

**Authors:** Aleksandr Kapitonov, Sergey Lonshakov, Vitaly Bulatov, Babak Kia Montazam, James White

**Affiliations:** M2M Economy, Inc (Merklebot), San Francisco, CA, United States

**Keywords:** robot economics, cloud criticism, autonomous agents, centralized and decentralized systems, Robot-as-a-Service

## Abstract

This article is devoted to the historical overview of the Robot-as-a-Service concept. Several major scientific publications on the development of Robot-as-a-Service systems based on a service-oriented paradigm are considered. Much attention is paid to the analysis of a centralized approach in the development using cloud computing services and the search for the limitations of this approach. As a result, general conclusions on the reviewed publications are given, as well as the authors' own vision of Robot-as-a-Service systems based on the concept of robot economics.

## 1. Introduction

The automation and robotization trend that began in the second half of the twentieth century is now moving into a qualitatively new stage. Due to the widespread adoption of the Internet, mobile devices, sensors, and video cameras, as well as deep learning methods, almost every corner of a large city becomes digitized (Lyons et al., 2018). The proliferation of unmanned aerial vehicles (Ortiz et al., 2018) and cheap and high-performance satellite Internet channels (Sayin et al., 2019) promises even more extensive digitalization, now on a global scale. Engineering also does not stand still, and in recent years, developers have achieved outstanding results that open up new opportunities for using robots in everyday life (Nelson et al., 2018).

These changes cannot but affect the most important sphere of human welfare—the economy. The development of digital technologies has led to “uberization” of the relationships between the client and the service providers (David et al., 2016). The as-a-service business model has completely dominated the software industry in the past several years. It has grown to other sectors now, where continuous recurring revenue has replaced the one-time purchases.

Immersive robotization paves the way for entirely new business models and concepts (Wirtz et al., 2018). This is not just about changes in the supply chain for large enterprizes due to the introduction of automatic lines. It is about universal access to robotic capabilities: from small and medium-sized businesses to individual use.

Historically, acquiring new equipment has most commonly been done through debt financing or leasing. Furthermore, bonds have been issued to finance previous industrial revolutions. As a society, we could finance national railroad systems, large-scale industrial manufacturing, and iconic infrastructure like Golden Gate Bridge.

These trends will affect how the 4th Industrial Revolution will unwrap. Connected and complex robotics systems create an opportunity for traditional equipment financing models to evolve and modernize, making them more in line with the as-a-service approach used in other industries.

However, appropriate architectures and principles of their work should be proposed before implementing such systems in the economy. Among other things, control systems of agents should address the following issues:

- How can a client understand what services this autonomous agent is capable of performing?
- How easy is it to integrate a new list of services?
- How can an agent coordinate its work with other devices or programs if the task is too tricky for it alone?
- How to keep records of agents, track their condition, and check the services' quality?
- How to solve the inevitable cybersecurity issues?

Researchers have already begun to search for solutions. The most famous concept is Robot-as a-Service (RaaS)—the concept of service-oriented architecture (SOA), which provides the integration of robots and devices into a single computing environment (Chen et al., 2010). This allows companies to avoid capital expenditures when acquiring new robots by creating an agreement with the robot provider. The payments will vary depending on the specific parameter, like time or number of operations.

RaaS largely explains how one can solve the problems of using autonomous agents to deliver services, but it has some nuances. In this article, we would like to look at the latest research on RaaS, identify the advantages and disadvantages of this concept, and suggest a possible development of this area.

The article is structured as follows. In section 2, we give a summary of the concept of RaaS. The entire section 3 will be devoted to the most prominent publications on this topic. In section 4, we give a general comment on the concept based on the publications' information. In section 5, we describe our vision for developing the RaaS concept. Economic reasons for using peering technologies are shown in section 6. The conclusion is summarized in the last section.

## 2. Robot-as-a-Service Concept

As already mentioned, the concept of Robot-as-a-Service appeared as part of a service-oriented paradigm, continuing the list of “as-a-Service” concepts (Platform-as-a-Service, Software-as-a-Service) (Blokdyk, 2018). In short, the hallmark of these concepts is the refusal to purchase hardware or software directly. Instead, it is proposed to receive all the necessary services by subscribing to them. RaaS involves the rental of robotic devices (often expensive) with the ability to deploy your applications and services.

**Figure 1:**
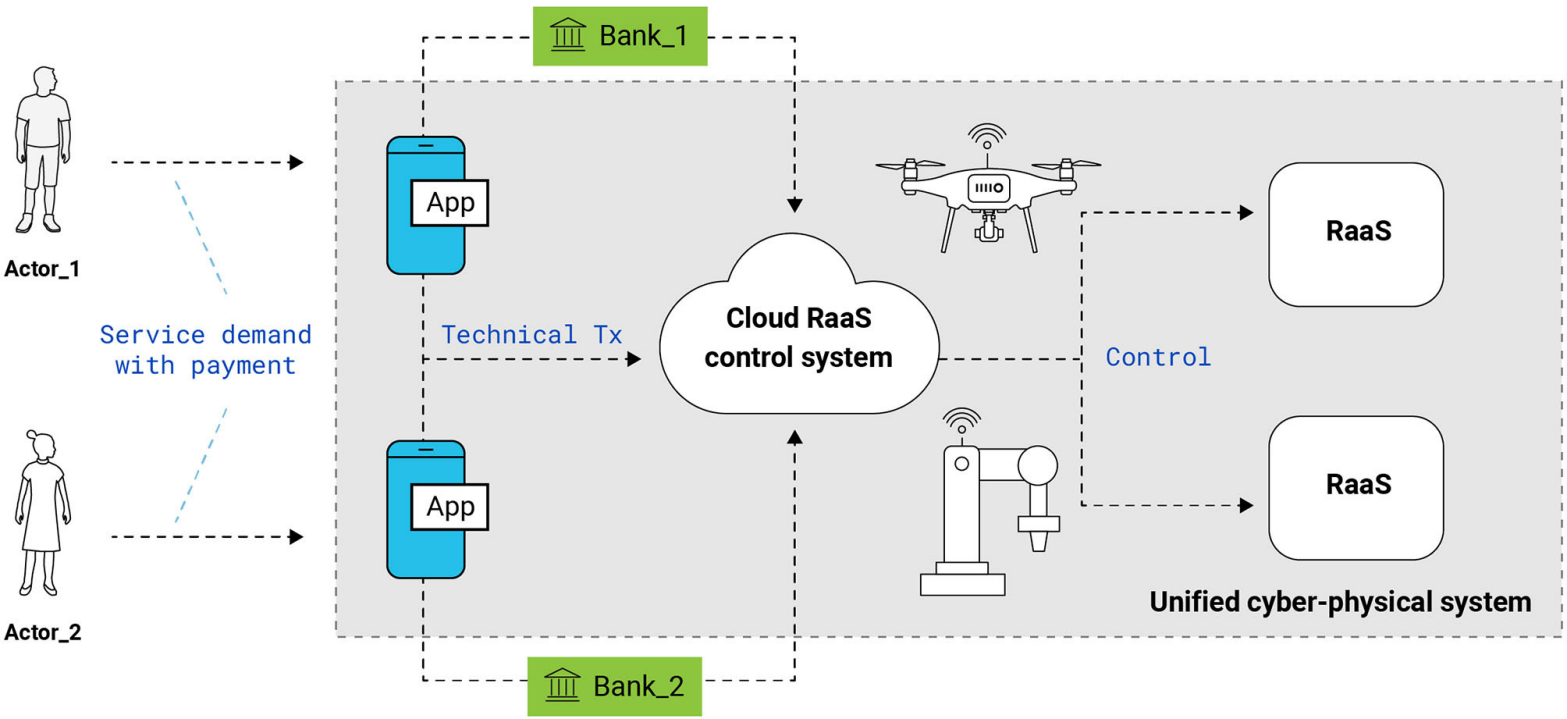
An example scheme of the RaaS architecture is based on cloud solutions. Actors (i.e., users) send a request for the provision of a service through the application. The request is then divided into an economic transaction (which is processed in a standard way through the banking system) and a technical transaction sent to the cloud. The cloud then sends command signals to the needed devices.

Typically, the RaaS platform contains: basic services that describe the functionality of the robot; the ability to add and select user services; standardized communication protocol (e.g., Web Services Description Language, Simple Object Access Protocol, HTTP); integration with a computing environment and a database for performing complex calculations and storing information (Blokdyk, 2018). The last item in the first and most popular implementation of RaaS is represented by cloud computing services (from Amazon, Google, Microsoft, and other enterprizes). An exemplary description of the RaaS system's operation through the cloud is shown in Figure 1.

In scientific publications, the robot-as-a-Service concept development is primarily associated with the works of Yinong Chen^1^ and his colleagues from Arizona State University. In the next section, we will discuss them in more detail.

## 3. Historical Overview of RaaS Publications

This overview was done by searching with the keyword for “robot-as-a-service” in scientific databases. The purpose of this review is to give an idea of how progress in this area. In addition to a general overview of the technology, we look primarily at how authors integrate their work into the economy.

### 3.1. Robot as a Service in Cloud Computing, 2010

The most cited publication on the RaaS concept and one of the first publications to use this term (Chen et al., 2010). For a long time, the authors were engaged in service-oriented architecture and cloud computing together with Intel and Microsoft, and at that time, it was logical to resort to the dominant SOA approach with respect to RaaS.

In their understanding, RaaS should have all the functions presented in SOA: service provider, service broker, service client. That is, for each device there is a repository with downloadable services, all services are available for the client in a shared directory, and a new application can be created based on existing ones.

As a result, the RaaS unit is a robot for which all the described functions are implemented, and access to them is in the cloud. The cloud is the provider, and the broker is an interactive shell that allows us to view and select any of the services. Interaction between units occurs through a standard interface (WiFi, Bluetooth), and between services through a Web Services Description Language. The authors devote much time to their RaaS prototype on Arduino, Intel, and Lego NXT boards and show how such an architecture can be ported to various platforms (Java on Linux, C#, and Visual Programming Language on Windows).

### 3.2. Internet of Intelligent Things and Robot as a Service, 2013

This article is from the same authors (Chen and Hu, 2013) and it presents a more extended concept of RaaS focused on the Internet of Things (IoT). The authors emphasize a critical feature of RaaS: they note the cyclical nature of the changes in centralized and decentralized paradigms for constructing such systems. Noting the disadvantages of decentralization, the authors nevertheless explain that decentralized elements are needed to increase the centralized system's reliability. Among the typical narrow points for RaaS systems, they present scalability, service orientation, security, adaptability to changes, and fault tolerance.

Authors propose increasing fault tolerance through standardization and redundancy. To do this, they suggest:

- Unified standard cloud access protocols.
- Standardized interfaces for interacting with the environment and end-users.
- Reserve cloud connectivity ports.
- Neighboring devices should form a mutually replacing structure: the nearest one takes the place of the failed one.

In addition, the authors discuss effective methods for modeling the behavior of devices in decentralized conditions. They note that the algorithms for solving context-sensitive and situational-sensitive tasks are too complex and slow for the Internet of Things and RaaS. The main reason: the difficulty of resolving conflict situations with the classical logic approach. Therefore, they propose using activity theory and expansive systems theory to solve this problem. The rest of the article is devoted to the implementation of RaaS test systems and experiments.

### 3.3. Robot Cloud: Bridging the Power of Robotics and Cloud Computing, 2017

This is the latest major publication from Du et al. (2017). In this work, the authors consider a more global structure—the multi-agent RaaS system, together with tight integration with cloud services that they call Robot Cloud. An important contribution of this work is expressed in a formal architectural scheme with various levels: perception, transport, mapping, processing, application, and business layers. Of particular note is the presence of a business layer—not only it manages the fee for using the services, but it also is searching for new business models that could contribute to the long-term development of the whole structure.

In Robot Cloud, a service broker is implemented in Cloud Robot Host—in a unit that controls the entire apparatus of robotic executors. The unit is responsible for authentication and registration of robots, monitoring their status, planning their work, finding violations, providing a list of services for users.

Then they formally describe the behavioral model of their service robots and simulate one large instance of their system, which serves an area of 20 × 14 km with a different number of robot units (from 100 to 400 units). A simple goal was chosen for this experiment: robots must come to a specific place and stop there for a while (for example, in reality, it can be a robotic show). The fee is charged depending on the service time, and the demand for robot services is continuously changing. Also, robots periodically require recharging.

Thanks to the simulation, they were able to compare the performance of only one major center and several smaller ones in order to find out which approach (centralized or decentralized) is better. In their case, creating one large center for solving a joint problem shows the best performance.

### 3.4. A Service-Oriented Architecture for Virtualizing Robots in Robot-as-a-Service Clouds, 2014

This publication continues the development of the previously described RaaS concept. Koubaa (2014) offers RoboWeb, a service-oriented architecture based on the Simple Object Access Protocol. An important contribution to this work is the use of the Robot Operating System (ROS) framework. Any information on robots' actions is published via ROS in the appropriate topics, making it easier to interact with services. ROS also allows to unify the interaction between robots and use a wide range of different robotic devices.

### 3.5. Robots as-a-Service in Cloud Computing: Search and Rescue in Large-Scale Disasters Case Study, 2018

This article (Mouradian et al., 2018) presents a specific implementation of RaaS for an emergency response system. Among the possible applications are: robotic sealing of leaks in a nuclear reactor, coordination of search and rescue operations during natural disasters, etc. The authors consider the robots' activity as a set of services, and the final solution to an emergency response task as a combination of these services by different robots. Accordingly, from this idea, the authors establish the requirements for the RaaS system: a standard and unified behavior model, mechanisms for publishing robot activity, a mechanism for jointly performing a common task, and a mechanism for evaluating its progress.

It is important to note how the authors describe the interaction interface between autonomous agents: it is very similar to the ROS publisher-subscriber system. Then the authors try to find out which existing control models can be suitable for RaaS and conduct several experiments on the Lego NXT, Google App Engine, and the Juxtapose P2P protocol.

## 4. Discussion

As we can see, when it comes to building Robot as-a-Service systems, the SOA concept based on cloud computing is practically the only one that is being researched and implemented. The difference is only in the details of the RaaS implementation, such as various communication protocols.

The overall architecture remains highly centralized, despite attempts to introduce decentralized elements. This is, in our opinion, a critical place for the implementation of RaaS systems. Studies show (Cummings, 2015) that decentralization reduces the computational burden on the agent management system and allows us to connect more devices than a centralized one. Moreover, large cloud services are highly centralized structures, which can adversely affect the security of RaaS systems. In the event of a center failure or hacking, the multi-agent system of robots will simply cease its activity at best. The worst-case scenario involves taking control of robots and devices and subsequent misconduct.

An interesting suggestion was made about the mechanism for detecting a faulty/hacked agent. It is proposed to compare the executable code between agents and determine the difference in behavior using the voting process. However, specific security issues are not raised among RaaS publications. Moreover, the issue of transparency of operations performed by agents is beyond the scope of these articles.

Most authors note that RaaS systems require unified interfaces and standardized communication protocols between agents since robots of various types and models can perform the common task. The Robot Operating System's appearance attracted noticeable attention to this framework: it is either successfully implemented as a communication protocol, or similar functionality is laid in the foundation of its own interaction mechanisms.

Another point that is hardly covered by the authors is the economic component of the RaaS system. Only one publication mentioned the business layer in the system architecture, which should be responsible for the economic behavior of agents and the collection of user fees. But this part of the system is most important in the context of implementing any business model. For successful RaaS-based business automation, robotic devices need to integrate the ability to conduct and accept economic transactions, because this is the only way to enable automated analytics on the effectiveness of business processes. In addition, a clear link between the effectiveness of running services and the cash flow of transactions allows for transparency between customers and service providers.

After reviewing these works, we can say that the future of the RaaS concept should be revised. Focusing only on cloud services leads to excessive centralization, lack of transparency and possible security problems with such systems.

## 5. Our Vision of RaaS Future

**Figure 2:**
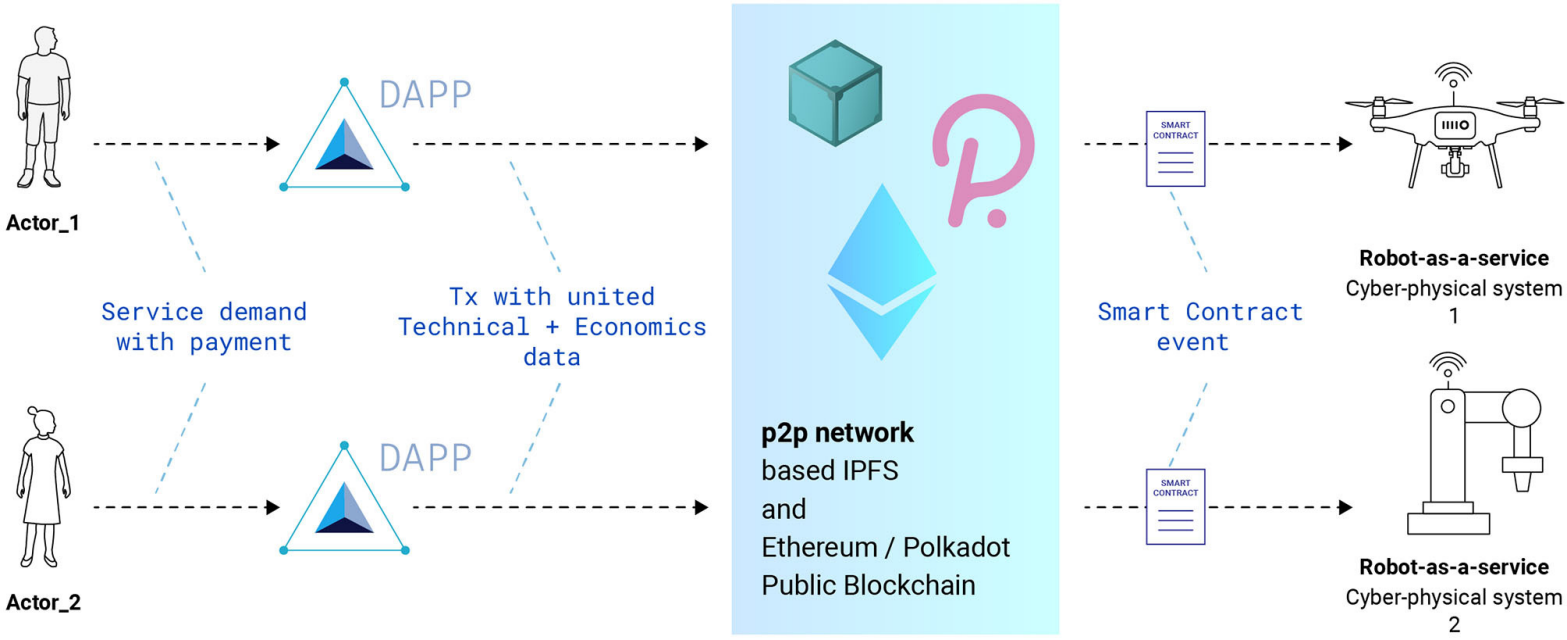
An example scheme of the RaaS architecture based on decentralized technologies. Actors send a request for a service through the decentralized application (Dapp). Economic and technical transactions are sent to a decentralized network and then they go directly thought smart contracts to a specific device.

**Figure 3:**
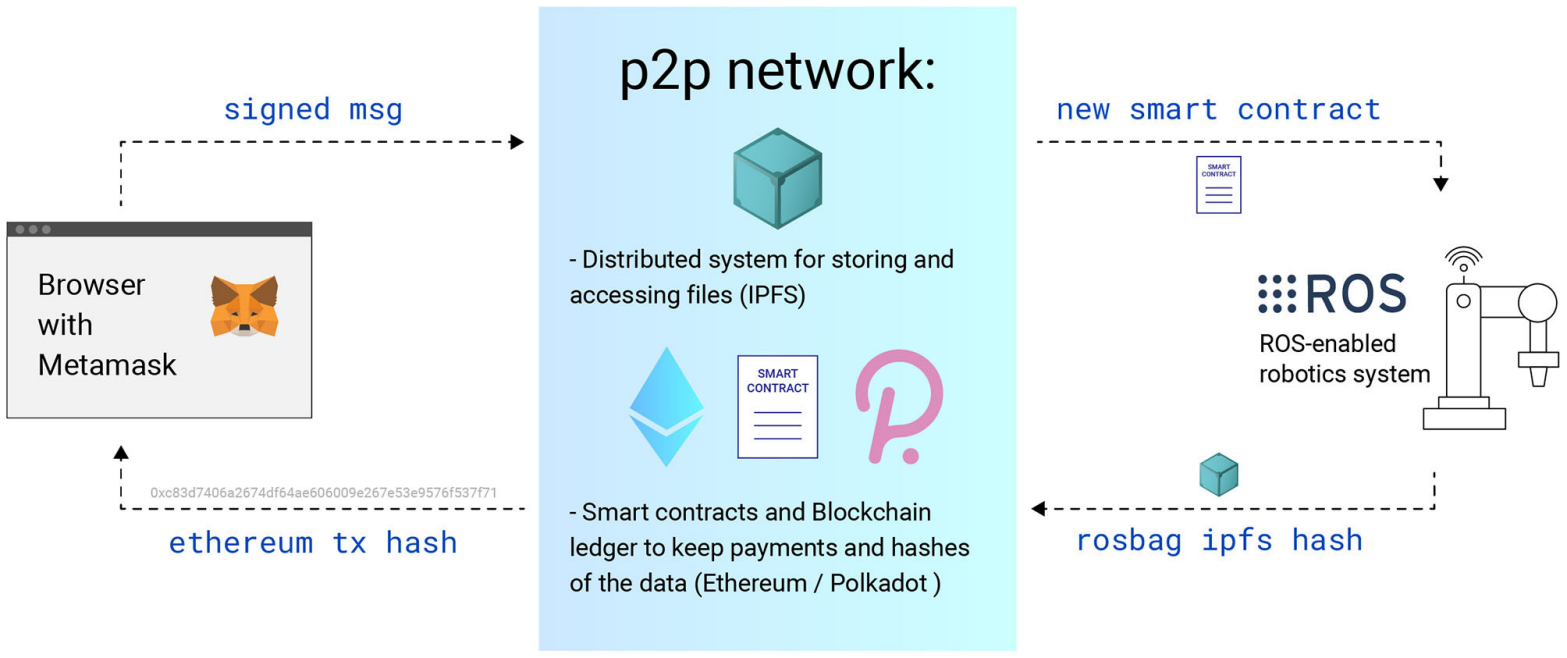
The scheme of work of our RaaS architecture. As a Dapp for ordering a service from a robot, a regular web browser with an Metamask extension for access to cryptocurrency wallets is used.

An alternative to the cloud-based, centralized approach to creating RaaS systems is a decentralized approach (Figure 2) based on p2p technologies (Kermarrec and Ta¨ıani, 2015; Schmitt et al., 2015; Afanasyev et al., 2019). We would like to offer our own vision of RaaS, based on the concept of robot economics (Kapitonov et al., 2019). In this concept, we offer a broad decentralization of robotic devices, giving them economic independence. This is proposed to be achieved through the use of p2p technologies, distributed registry, and cryptography (Dorigo et al., 2018; Ferrer, 2018). The Figure 3 shows the architecture of our Robot-as-a-Service vision.

The following is a short list of characteristics of the robot economics:

- The work of agents is organized according to market principles—messages are published on special channels about the demand for certain robotic services and supply offered by robotics in the network.
- Economic transactions between robotic agents and users, as well as between agents themselves, are carried out using smart contracts in one of the distributed registries (Ethereum, Tezos, Polkadot/Substrate). Thanks to smart contracts technology, the execution of robots' code is guaranteed while maintaining high standards of protection against malfunctions and hacking.
- By using a distributed registry, the agents can transfer value and each agent can have its own wallet to perform transactions. This opens up the possibility of using different optimization algorithms either between many partners in the supply chain or even among the different divisions of a complex organization.
- Also, a distributed registry allows for transparency of transactions between nodes. The functions of the cloud center are transferred to software nodes that are as independent as the robots. This is beneficial for both internal accountability and external auditing.
- The content-addressing distributed file system (InterPlanetary File System) is used to store data and to publish supply/demand. By storing the hashes of the data in distributed ledger full data immutability is achieved.
- The unified communication protocol for various devices is Robot Operating System.

In Table 1, we have compiled a short comparison between cloud-based and decentralized solutions for RaaS in terms of communication speed, transaction costs and operational security for robots. For comparison, we mentioned three of the most promising decentralized technologies in our opinion, which can become powerful foundations for Robot-as-a-Service module: InterPlanetary File System^2^, Ethereum^3^ (and its future version^4^) and Polkadot^5^.

**Table 1:** Comparison between cloud and decentralized technologies for RaaS.

**Parameter**	**Cloud**	**IPFS + Ethereum**	**IPFS + Eth2.0/Polkadot**
App-to-RaaS communication speed	High or medium	Low, ~each 15 s	Medium, each 2–4 s
Transaction cost	High, <20$ + fees	Medium, ~0.1–0.2$	Potentially low
Robot launch security	Low	High	High or medium?

If we talk about communication speed, the most important metric for cloud is IoT connectivity bandwidth and execution speed of banks payment gateway transaction (for example, Visa system processes around 1,700 transactions per second) which by current days can be evaluated as high. For comparison Ethereum 1.0 produces blocks around each 15 s with a limited number of transactions in block. However, Ethereum 2.0 and Polkadot theoretically can produce blocks each 2–4 s with unlimited number of transactions.

Average price of cloud server for IoT connectivity starting less 20$ and a bank payment fee. Also RaaS Provider have a cost to keep records. These factors increase the transaction costs of deploying a large structure with many robots and transactions. On the other hand, decentralized technologies are deprived of the need for an expensive central intermediate. Transactions cost for the creation of a new smart contract in Ethereum public blockchain starts from 0.1−0.2$. Moreover, based on sharding and parachain concept we can expect much lower price (Dang et al., 2019; Schulte et al., 2019).

At the same time, cloud technologies are at a greater risk of security. An additional payment flow with bank intermediaries creates security risks in the launch process. Launching robots under cloud control creates a single point of failure in the architecture of the system. Moreover, there is no transparency inside the cloud, data, and any evidence can be deleted at any time. Decentralized technology, based on its basic principle, is designed to solve these problems. Consensus based decentralized computer, Ethereum, has never been shut down after its launch in the summer 2015. It has high level of transparency of what happens with user requests after the user sent transaction. Immutable and infinity lifetime of records in blockchain also increases its reliability. However, with the current Ethereum, there is a trilemma between decentralization, scalability, and security: such systems can only at most have two of three properties. How this problem will be solved in the future is an open question^6^.

## 6. Economic Reasons for Using Peering Technologies

It is important to note that the introduction of anything as a service model (XaaS) has emerged with the advent of cloud infrastructure but continues to evolve further.

First, let's outline the benefits that the XaaS model brings to the robotics market. The idea of “as a service” model allows businesses to reduce the costs of using and deploying robotic devices. Before the introduction of a service model, companies had to purchase the equipment, bear integration and maintenance costs. They had to buy constant updates and expand their networks on their own. Finally, they had to worry about the security of their network.

With the introduction of the RaaS model companies can get access to robotic devices without the need to maintain their own infrastructure. This allows unlocking the next phase of robotics technology adoption as RaaS significantly reduces the cost of deploying robotic devices by replacing the large initial capital expenses for more consistent, smaller regular payments. More small and medium businesses can now use robotics in their facilities, and many more use cases become possible. RaaS business model is not just about changes due to the introduction of automatic lines. We are talking about universal access to robotic capabilities for everybody from small and medium-sized businesses to even individual use.

This paper describes the infrastructure and tools that help companies to connect robots to the internet. But in contrast with the most common approaches discussed above which use cloud infrastructure, we chose to focus on technologies like Ethereum and IPFS, which have two significant benefits that are unique to this stack.

First, these technologies allow direct human to machine communication without cloud connection. Oftentimes, cloud connection can reduce communication speed and affect latency, which can be crucial in many industrial applications. Moreover, the use of these technologies can allow to solve security issues. By default, the platforms that were proposed have built-in security instruments like public key cryptography and hashing algorithms that protect the communication and data.

Secondly, this set of technologies allows to create a fully autonomous machine to machine economy where robots and algorithms can exchange both technical data (like sensor metrics and robot commands) and economic data (like payments) in a single transaction. Thanks to blockchain technology we can combine technical and economic data in a single transaction to achieve more efficient and secure communication, which, in turn, allows us to organize large and complex supply chains fully autonomously.

RaaS concept is going to change the robotics market and peering technologies enable more secure, transparent, and efficient communication that combines both economic and technical aspects.

## 7. Conclusion

In this article, we have led a guided tour of the nascent sphere of Robot-as-a-Service. As can be seen from the overview of the most popular and well-known publications in this area, now this concept is fixated on service-oriented architecture based on cloud technologies. Cloud technologies have a number of critical limitations for this concept, and in our opinion the lack of alternatives to this approach only inhibits the development of commercial robotics. That is why we turned to decentralized technologies and presented our vision of the future RaaS. In our case, the foundation of the architecture is distributed registry technology and distributed file systems combined with the most powerful frameworks for robotic systems.

In future work, we will study the effectiveness of RaaS systems built on such principles as compared to cloud systems, for which it is proposed to develop large-scale simulations of such a ystem.

## Author Contributions

All authors listed have made a substantial, direct and intellectual contribution to the work, and approved it for publication.

## Conflict of Interest

All authors were employed by the company M2M Economy Inc (Merklebot).

## Footnotes

^1^Personal page of Yinong Chen—http://www.public.asu.edu/~ychen10/.

^2^IPFS Powers the Distributed Web, URL: https://ipfs.io/.

^3^Ethereum—a global, open-source platform for decentralized applications, URL: https://ethereum.org/.

^4^Ethereum 2.0 Specifications, URL: https://github.com/ethereum/eth2.0-specs.

^5^Polkadot: Decentralized Web 3.0 Blockchain Interoperability Platform, Web: https://polkadot.network/.

^6^On sharding blockchains, URL: https://github.com/ethereum/wiki/wiki/Sharding-FAQ.
